# Assessing Factors Related to Waist Circumference and Obesity: Application of a Latent Variable Model

**DOI:** 10.1155/2015/893198

**Published:** 2015-12-03

**Authors:** Sahar Dalvand, Jalil Koohpayehzadeh, Masoud Karimlou, Fereshteh Asgari, Ali Rafei, Behjat Seifi, Seyed Hassan Niksima, Enayatollah Bakhshi

**Affiliations:** ^1^Department of Biostatistics, University of Social Welfare and Rehabilitation Sciences, Tehran 1985713834, Iran; ^2^Center for Diseases Control, Ministry of Health and Medical Education, Tehran, Iran; ^3^Department of Physiology, School of Medicine, Tehran University of Medical Sciences, Tehran, Iran; ^4^Department of Epidemiology and Biostatistics, School of Public Health, Tehran University of Medical Sciences, Tehran, Iran

## Abstract

*Background*. Because the use of BMI (Body Mass Index) alone as a measure of adiposity has been criticized, in the present study our aim was to fit a latent variable model to simultaneously examine the factors that affect waist circumference (continuous outcome) and obesity (binary outcome) among Iranian adults.* Methods*. Data included 18,990 Iranian individuals aged 20–65 years that are derived from the third National Survey of Noncommunicable Diseases Risk Factors in Iran. Using latent variable model, we estimated the relation of two correlated responses (waist circumference and obesity) with independent variables including age, gender, PR (Place of Residence), PA (physical activity), smoking status, SBP (Systolic Blood Pressure), DBP (Diastolic Blood Pressure), CHOL (cholesterol), FBG (Fasting Blood Glucose), diabetes, and FHD (family history of diabetes).* Results*. All variables were related to both obesity and waist circumference (WC). Older age, female sex, being an urban resident, physical inactivity, nonsmoking, hypertension, hypercholesterolemia, hyperglycemia, diabetes, and having family history of diabetes were significant risk factors that increased WC and obesity.* Conclusions*. Findings from this study of Iranian adult settings offer more insights into factors associated with high WC and high prevalence of obesity in this population.

## 1. Introduction

Obesity has emerged as a public health problem worldwide. Indeed, it is now so common that it is replacing the more traditional public health concerns (i.e., under nutrition and infectious diseases) [1]. The prevalence of obesity has increased in the last century and was dramatically accentuated in recent decades. While Body Mass Index (BMI) has been shown to predict abdominal fat and abdominal subcutaneous fat, waist circumference has been shown to predict visceral fat, thus reinforcing the use of both BMI and waist circumference in clinical practice. An operational definition of obesity, useful in many contexts, is BMI ≥ 30.0 kg/m^2^, but it should not be used as a conceptual definition. The obesity denotes excess body fat in a large amount, enough to cause a reduction in health or longevity. This health impairment will not be visible in all cases according to the operational definition used, but the risks caused by obesity impair future health [2]. Measuring waist circumference helps screen for possible health risks that come with overweight and obesity. According to the estimates of the World Health Organization (WHO), overweight and obesity are the fifth risk factor for global deaths, causing approximately 2.8 million deaths per year [3]. Prevalence estimates for overweight and obesity reach 1.4 billion adults over 20 years, obesity alone covering over 10% of world population [4]. The prevalence of obesity has doubled or even risen threefold in less than two decades [5]. Now, Iran faces growing challenges of aging and noncommunicable diseases. The rising healthcare cost of the estimated number of population with obesity around the world is 1.5 billion in 2012 and it continues to rise [6]. It is estimated that, by the year 2015, 2.3 billion people will be overweight and 700 million will be obese [7]. The prevalence of obesity in USA, Canada, Australia, United Kingdom, Iran, and Egypt was 31.8, 24.3, 25.1, 24.9, 21.6, and 34.6, respectively [8]. According to the United States Department of Health and Human Services (HHS) the following individuals are at increased risk for developing chronic diseases: women with a waist circumference of more than 89 centimeters (35 inches) and men with a waist circumference of more than 102 centimeters (40 inches). However, lower thresholds for waist circumference have been recommended for Asian populations by WHO due to recent research findings. Therefore, those at increased risk for developing chronic disease include Asian women with a waist circumference of more than 79 centimeters (31 inches) and Asian men with a waist circumference of more than 89 centimeters (35 inches) [9]. At the same BMI, Asians tend to have a higher percent of body fat and more central adiposity [10]. Department of WHO reported that obesity is increasing in the world [1] and it is also an established health problem in Iran [11]. The top 10 obesity-related diseases include high blood pressure, diabetes, heart disease, high cholesterol levels, cancer, infertility, back pain, skin infections, ulcers, and gallstones [12]. In 2002 about 41,000 new cases of cancer in the United States were estimated to be due to obesity. This means that about 3.2 percent of all new cancers are linked to obesity [13]. In developing countries, increasing overweight and obesity have been reported with greater degree of urbanization [14–17]. Smoking and obesity are leading causes of morbidity and mortality worldwide [18, 19]. It is widely accepted that hypertension is more common among the obese than among the lean and that a positive relationship exists between the level of blood pressure and the degree of obesity [20]. It is estimated that at least 75 percent of the incidence of hypertension is related directly to obesity [21]. Family history of diabetes has been recognized as an important risk factor of the disease [22]. In 2005 the estimated total numbers of worldwide obese and overweight adults were 396 million and 937 million, respectively. The numbers have differed by race, gender, and geographic location [23]. Apart from medical factors, researchers have focused on nonmedical factors such as sociodemographic and lifestyle factors. Age, sex, physical activity, and socioeconomic state have shown a relation to obesity [24–29]. In the present study our aim was to fit a latent variable model to simultaneously examine the factors that affect WC (continuous outcome) and obesity (binary outcome) among Iranian adults and to provide health professionals with appropriate weight-management guidelines for this population.

## 2. Materials and Methods

### 2.1. Data Source

Data for the present study were derived from the third round of the survey of Noncommunicable Diseases Risk Factors Surveillance in Iran. This population-based cross-sectional study was conducted by Iran Center for Diseases Control. A cluster sampling design was used to produce representative data for that age range in Iran. The number of clusters in each province was proportional to the size of that province, each cluster comprising 10 men and 10 women. For each province, a total of 50 clusters including 20 participants, two males and two females in each 10-year age group, were selected using a proportional-to-size systematic sampling scheme. The households addresses were extracted by Iran's Post Company. Eventually, participants were interviewed at their homes after receiving an informed consent by trained healthcare workers. Based on the STEP-wise approach of WHO, STEPS is a sequential process, starting with gathering information on key risk factors by the use of questionnaires (Step 1), then moving to simple physical measurements (Step 2), and only then recommending the collection of blood samples for biochemical assessment (Step 3) [30]. Participants were interviewed at their homes by trained healthcare workers from 43 medical schools and a blood sample was taken after receiving a verbal informed consent. After excluding pregnant women, the data analyzed included 18,990 women and men aged ≥20 years.

### 2.2. Measurements and Variables

Interview phase of the study was performed using a standard questionnaire measuring demographic, behavioral, and physical risk factors proposed by WHO.

### 2.3. Response Variables

Waist circumference (WC) and obesity were treated as the main response variables of the study.

*WC (Continuous Outcome).* It is a measure of the distance around the abdomen in centimeter.

*Obesity (Binary Outcome).* People who are obese have an abnormally high and unhealthy proportion of body fat. To measure obesity, researchers commonly use a formula based on weight and height known as BMI. According to the World Health Organization (WHO), a measurement obtained by dividing a person's weight by the square of the person's height exceeds 30 kg/m^2^.

### 2.4. Independent Variables

*Age (Year).* Information about the respondent's age was based on their self-reported birth year. Adults were stratified into five 10-year age groups (20–29, 30–39, 40–49, 50–59, and 60+ years).

*PA.* Physical activity is any body movement that works your muscles and requires more energy than resting. PA was a composition measure of different activities as queried in the questionnaire, and participants were stratified into three groups (low/moderate/heavy).

*Smoking Status.* Smoking status was dichotomized into smokers versus nonsmokers.

*Blood Pressure.* Normal blood pressure at rest is within the range of 100–140 mmHg systolic (top reading) and 60–90 mmHg diastolic (bottom reading). High blood pressure is said to be present if it is often at or above 140/90 mmHg.

*CHOL.* Cholesterol, according to sources in the hospital laboratory cut-off point of 200 (cholesterol ≥200 indicates hypercholesterolemia and cholesterol <200 is desirable cholesterol), is used.

*FBG.* Fasting Blood Glucose, as the name suggests, means refraining from eating or drinking any liquids other than water for eight hours. The respondents were categorized into two groups. Adults who had glucose level more than 126 mg/dL were considered hyperglycemia; others were considered normal FBG.

### 2.5. Method

#### 2.5.1. Overview of Latent Variable Model

According to the method proposed by Teixeira-Pinto and Normand [31] it is assumed that *y*_*b*_ and *y*_*c*_ are binary and continuous response variable associated with covariates *x*_*b*_ and *x*_*c*_. The variable *y*_*c*_ is assumed to be normally distributed given the covariate *x*_*c*_. They developed a multivariate model that takes into account the potential correlation between *y*_*b*_ and *y*_*c*_. Suppose there is an underlying variable *y*_*bi*_^*∗*^, normally distributed given the covariates *x*_*bi*_, that is associated with the binary outcome, *y*_*bi*_, in the following way:

(1) $$\begin{array}{c} y_{bi}=\left\{\begin{array}{cc} 0, & \text{if }y_{bi}^{*}\leq 0\\ 1, & \text{if }y_{bi}^{*}>0. \end{array}\right. \end{array}$$

We can write the regression equation for the binary outcome, *y*_*bi*_, as *P*(*y*_*bi*_ = 1∣*x*_*bi*_, *u*_*i*_) = *P*(*y*_*bi*_^*∗*^ > 0∣*x*_*bi*_, *u*_*i*_) = Φ(*x*_*bi*_^*T*^ *β*_*b*_^*∗*^ + *u*_*i*_), where Φ(·) represents the cumulative distribution function (cdf) of the standard normal distribution. Probit link function is used for binary response variable. The regression equation for two response variables is written as follows:

(2) probitPybi=1 ∣ xbi,ui=XbiTβb∗+ui,yc ∣ xci,ui=XciTβc+σcui+ϵci.

That is, *ϵ*_*ci*_ ~ *N*(0,1) and the latent variable *u*_*i*_ ~ *N*(0, *σ*_*u*_^2^).

### 2.6. Statistical Analysis

WC was treated as a continuous outcome variable and obesity as binary outcome variable. The parameters *β*_*b*_^*∗*^ in (2) are interpreted as conditional effects on *u*_*i*_. For this reason the parameters *β*_*b*_^*∗*^ of the latent model cannot be directly compared with the regression parameters of the marginal models. So, estimates for the marginal effects $\hat{\beta_{b}}$ are obtained using $\beta_{b}=\beta_{b}^{*}/\sqrt{1+\sigma_{u}^{2}}$. For the continuous outcome, *β*_*c*_ is interpreted as conditional or marginal effects of the covariates. Analyses results were obtained using SAS, version 9.2.

## 3. Results

Distributions of covariates are shown in Table 1 to make the data presentation complete. Our results showed that the prevalence of obesity was higher among older age groups, females, nonsmokers, and those residing in urban areas. Obesity is frequently observed in people with hyperglycemia, high blood pressure, high cholesterol, and lower PA. About 51.9% of participants were females and 48.1% were males. The majority of the sample was between the ages of 30 and 59 years (76.2%). Urban residents had higher obesity prevalence rates (26.1%) than rural residents (18.3%). It was illustrated that nearly 37.7%, 23.6%, and 38.7% of people had low, moderate, and heavy level of PA, respectively. Nearly 7.4% and 22% of participants had diabetes and family history of diabetes, respectively. Almost 7% of people had FBG level more than 126 mg/dL. Nearly 21.5% of participants had SBP more than 140 mg/dL and 21.1% of participants had DBP more than 90 mg/dL. Nearly 37.7% were hypercholesterolemia and 14.5% of participants were smokers. Women were more likely to be obese (31.1%) compared to men (13.6%).

Results in Table 2 are obtained from fitting latent variable model based on the 18,990 Iranian adults. Among adults, age, being inactive, being an urban resident, being nonsmoker, and being female were directly associated with WC and obesity. Obesity and WC are also directly associated with hyperglycemia, high blood pressure, and high cholesterol. In this sample for continuous outcome (WC), our results show that WC increase to 4.25, 6.79, 7.51, and 6.80 cm for age groups of 30–39, 40–49, 50–59, and 60+ years compared with the age group of 20–29, respectively. The mean of WC in females is 0.75 cm more than males and in urban people is 2.94 cm more than rural people. By using heavy level of PA as the reference group, WC increase to 0.84 cm for low level of PA. Using smokers as the reference group, the mean of WC increases to 3.10 cm for nonsmokers. The mean of WC increases to 2.16 and 2.82 cm among people with high level of SBP and DBP, respectively. In adults with high level of CHOL, the mean of WC increases to 3.66 cm compared with normal group. Among adults with hyperglycemia, the mean of WC increases to 2 cm compared to others. The mean of WC for adults with diabetes and FHD increase to 3.11 and 1.97 cm, respectively.

Our results in Table 2 also show that, for binary outcome (obesity), adults aged 30–39, 40–49, 50–59, and 60+ years increase the standard normal coefficient of obesity by 0.62, 0.66, 0.63, and 0.58, respectively, compared with age group of 20–29. Female sex increases the standard normal coefficient of obesity by 0.47. Our results show that residency in an urban area increases the standard normal coefficient of obesity by 0.41. Having a low level of PA increases the standard normal coefficient of obesity by 0.52 compared with heavy level. Being nonsmoker increases the standard normal coefficient of obesity by 0.61. Adults with high level of SBP, DBP, CHOL, FBG, and FHD increase the standard normal coefficient of obesity by 0.12, 0.27, 0.28, 0.09, and 0.19, respectively.

## 4. Discussion

In this national survey, we assessed associations between varieties of medical and nonmedical factors with WC and obesity in 18990 adults (aged ≥20 years) in Iran by using latent variable model. In our study, positive association was found between increased obesity and older age. Furthermore, the mean of WC increased with increasing age but the standard normal coefficient of obesity went up among adults aged 20–49 years and then it decreased for ages above 50 years. Changes in food intake, energy expenditure, appetite, and body composition that occurs with ageing could be related to the effect of age on obesity [32]. Different studies showed that overweight and obesity were associated with increased age in which there are hormonal changes and a common decrease in physical activity [33–35]. Although obesity prevalence increased in the world, there were differences between men and women in some regions and countries. Almost in all studies in Iran the joint frequency of obesity in women was more than men in all ages [36]. Generally, 13.6% of men in this study were obese, a statistic similar to reports from another study of Iran [37]. Gender differences were also present in this analysis for the adult Iranian population. Women were more likely to be obese than men. Statistically significant differences were found between adult men and women. Similar findings have been reported by others [36, 38–42]. Factors related to lifestyle may therefore be the reason for the high prevalence of obesity in women [40, 43]. Our results showed the differences between obesity and WC in urban and rural people. This could be a marker of bigger differences in urban/rural lifestyles in Iranian population, due to the recent acceleration of urbanization in Iran. Our findings did not agree with a study of ten European countries, in which no significant differences between urban and rural areas regarding obesity were detected in 9 of the 10 countries examined [15]. This etiology is multifactorial with genetic influences and environmental, socioeconomic, and behavioral and/or psychological causes playing a significant role and a relative increase in both WC and obesity. In line with some study, our results showed an inverse relationship between physical activity and obesity. Lack of PA and a hyper caloric nutrition are the main reason for obesity. PA is useful to burn calories and keep the muscular mass and increase the PA again [44]. So, most public health interventions attempt to create a negative energy balance by increasing PA. Rural residence in developing countries may be associated with more physical labor than urban settings and possibly obesity has lower prevalence in rural areas [14, 16, 45]. The relation between smoking and obesity is incompletely understood. Although most studies have indicated that smokers on average have lower body weights [46, 47], a few have reported that smoking and body weight are positively correlated [48]. Nicotine acutely increases energy expenditure and could reduce appetite, which likely explains why smokers tend to have lower body weight than nonsmokers and why smoking cessation is frequently followed by weight gain. Waist circumference (WC) is an indicator of the amount of visceral adipose tissue (VAT). A greater amount of VAT is related to the metabolic syndrome, diabetes, and cardiovascular diseases [49]. Cross-sectional studies indicate that WC is higher in smokers compared with nonsmokers [50–54]. Our results on the association between smoking and obesity are basically in agreement with those of other studies [24, 55]. Data from the Honolulu Heart Program [56] and the Japanese Data Bank Survey [57] indicate that obesity and high blood pressure continue to be correlated, even in old age. Obesity is associated with an increased risk of cardiovascular disease, but this requires that obesity is combined with hypertension. In obese subjects, the cardiovascular risk is not significantly increased unless hypertension is present [58]. The current study showed that individuals with the highest waist circumference quartile had 2-fold increased risk for hypertension compared with individuals with the lowest quartile [59]. We found a positive relationship between WC and obesity with hypertension. Both Framingham and Tecumseh studies have shown that future weight gain is significantly greater in hypertensive patients than in normotensive subjects [60, 61]. Many studies have shown that weight loss is effective in lowering the blood pressure [62–64]. Some studies showed that individuals with higher BMI have 14% chance of hypercholesterolemia [65]. Hypercholesterolemia is frequently found in patients with obesity, so that the average serum cholesterol level is significantly higher in overweight subjects than in lean ones, and usually a significant correlation exists between total cholesterol and obesity. The WC and possibly other body size measurements were independently related to the risk for high cholesterol, even among nonobese subjects [66]. Gostynski et al. [67] reported a strong positive association between hypercholesterolaemia and BMI. This finding corresponds well with the observations made in other cross-sectional studies, for example, the LRC Program Prevalence Study [68]. Our results are consistent with the hypothesis that with increase in obesity/WC the risk of hypercholesterolemia acutely increases. The present analysis had also revealed a positive association between WC and obesity with FHD and FBG. Okosun et al. [69] assessed the association of WC and risk of hypertension and type 2 diabetes in populations from several different African origins. They found that the higher categories of waist circumference were associated with larger excess in the prevalence of hyperglycemia in the presence of a family history of diabetes. If the association between obesity and hyperglycemia is different in individuals with a parental history of diabetes, this may affect decisions about weight reduction and screening for diabetes [70]. Although the reason for this difference is unclear, differences in other lifestyles or race might be important factors. These findings were in an agreement with some study [71].

### 4.1. Strengths and Limitations

A major strength of this study is that it included data from the Iranian population and the findings are applicable to populations in Asian countries. Despite this, because the use of BMI (Body Mass Index) alone as a measure of adiposity has been criticized, we used a latent variable model to simultaneously examine the factors that affect WC and obesity. We adjusted our analyses for a number of putative confounders including medical and nonmedical factors.

Consideration must be given to the potential limitations of this study, including its cross-sectional approach. We cannot establish a causal association between factors and obesity and WC or the direction of association. Although we adjusted our analyses for confounders, we have not included in our model other factors associated with obesity, such as marital status, dietary consumption, family income, and genotype because of the lack of information on these variables in this study.

## 5. Conclusion

In recent decades, risk factors for preventing obesity have been found. Obesity is now growing at an alarming rate reaching epidemic proportions worldwide thus increasing morbidity and mortality rates for chronic disease. Based on our findings, the lower prevalence of obesity among physically active Iranians was expected. As demonstrated, in adults obesity is real and we believe that this is belated time to create a healthy lifestyle. Unhealthy diet, physical inactivity, excess weight, and diabetes are taken into account as major causes of obesity. Findings from this study of Iranian adult settings offer more insights into factors associated with high WC and high prevalence of obesity in this population.

## Figures and Tables

**Table 1:** Descriptive characteristics of waist circumference and obesity across study variable levels.

Variables	Waist circumference		Obesity			*P* value
	Mean	SD	No	Percent	Total	
Age						
20–29	82.86	12.36	254	11.5	2206	<0.001
30–39	87.56	12.66	926	20.2	4584	
40–49	91.22	13.26	1284	26.3	4888	
50–59	93.04	13.43	1312	26.2	5006	
60+	92.85	13.63	532	23.1	2306	
Gender						
Male	89.52	12.90	1245	13.6	9130	<0.001
Female	90.53	14.06	3063	31.1	9860	
PR						
Urban	91.66	13.39	2792	26.1	10706	<0.001
Rural	87.96	13.41	1516	18.3	8284	
Physical activity						
Low	91.02	14.03	1919	26.8	7159	<0.001
Moderate	90.41	13.15	1028	23.0	4475	0.061
Heavy	88.87	13.15	1361	18.5	7356	<0.001
Smoking						
Nonsmoker	87.27	13.03	297	10.8	2760	<0.001
Smoker	90.52	13.55	4011	24.7	16230	
SBP						
Normal	88.66	13.24	3031	20.3	14906	<0.001
High	95.11	13.33	1277	31.3	4084	
DBP						
Normal	88.71	13.26	2951	19.7	14975	<0.001
High	95.03	13.33	1357	33.8	4015	
CHOL						
Normal	87.94	13.31	2143	18.1	11825	<0.001
High	93.52	13.16	2165	30.2	7165	
FBG						
Normal	89.54	13.42	3850	21.8	17672	<0.001
High	96.79	13.03	458	34.7	1318	
Diabetes						
Yes	97.23	12.02	499	35.4	1409	<0.001
No	89.47	13.47	3809	21.7	1758	
FHD						
Yes	92.68	13.24	1207	29.6	4076	<0.001
No	89.32	13.51	3101	20.8	14914	

**Table 2:** Estimates, standard errors, and *P* values of the association between factors and waist circumference and obesity obtained from latent variable model for Iranian adults.

Variables	Waist circumference		*P* value	Obesity			*P* value
	Estimate	SE		Estimate	SE	SN^*∗∗*^	
Age							
20–29	*∗∗∗*			*∗∗∗*			
30–39	4.25	0.32		0.31	0.04	0.62	
40–49	6.79	0.33	<0.001	0.42	0.04	0.66	<0.001
50–59	7.51	0.33		0.32	0.04	0.63	
60+	6.80	0.39		0.19	0.05	0.58	
Gender							
Female	0.75	0.21	<0.001	0.47	0.02	0.68	
Male	*∗∗∗*			*∗∗∗*			<0.001
PR							
Urban	2.94	0.19	<0.001	0.25	0.02	0.41	<0.001
Rural	*∗∗∗*			*∗∗∗*			
PA							
Low	0.84	0.22	<0.001	0.060	0.02	0.52	0.02
Moderate	0.24	0.70	0.07	0.007	0.03	0.50	0.79
Heavy	*∗∗∗*			*∗∗∗*			
Smoking							
Smoker	*∗∗∗*			*∗∗∗*			
Nonsmoker	3.10	0.28	<0.001	0.27	0.04	0.61	<0.001
SBP							
Normal	*∗∗∗*			*∗∗∗*			
High	2.16	0.29	<0.001	0.12	0.03	0.55	<0.001
DBP							
Normal	*∗∗∗*			*∗∗∗*			
High	2.82	0.28	<0.001	0.27	0.03	0.61	<0.001
CHOL							
Normal	*∗∗∗*			*∗∗∗*			
High	3.66	0.19	<0.001	0.28	0.02	0.61	<0.001
FBG							
Normal	*∗∗∗*			*∗∗∗*			
High	2.00	0.41	<0.001	0.09	0.04	0.54	0.03
Diabetes							
Yes	3.11	0.41	<0.001	0.16	0.04	0.44	<0.001
No	*∗∗∗*			*∗∗∗*			
FHD							
Yes	1.97	0.23	<0.001	0.19	0.03	0.43	<0.001
No	*∗∗∗*			*∗∗∗*			

^*∗∗*^Standard normal coefficient.

^*∗∗∗*^Comparator group.

## Abbreviations

WC: Waist circumference
PR: Place of Residence
PA: Physical activity
SBP: Systolic Blood Pressure
DBP: Diastolic Blood Pressure
CHOL: Cholesterol
FBG: Fasting Blood Glucose
FHD: Family history of diabetes
NCD: Noncommunicable disease
WHO: World Health Organization.
